# Increasing the penetration depth of temporal focusing multiphoton microscopy for neurobiological applications

**DOI:** 10.1088/1361-6463/ab16b4

**Published:** 2019-04-25

**Authors:** Christopher J Rowlands, Oliver T Bruns, Daniel Franke, Dai Fukamura, Rakesh K Jain, Moungi G Bawendi, Peter T C So

**Affiliations:** 1 Department of Bioengineering, Imperial College London, London SW7 2AZ, United Kingdom; 2 Helmholtz Pioneer Campus (HPC), Helmholtz Zentrum München, 85764 Neuherberg, Germany; 3 Department of Chemistry, Massachusetts Institute of Technology, Cambridge, MA, United States of America; 4 Edwin L. Steele Laboratory for Tumour Biology, Massachusetts General Hospital, Boston, MA, United States of America; 5 Harvard Medical School, Cambridge, MA, United States of America; 6 Department of Biological Engineering, Massachusetts Institute of Technology, Cambridge, MA, United States of America; 7 Department of Mechanical Engineering, Massachusetts Institute of Technology, Cambridge, MA, United States of America; c.rowlands@imperial.ac.uk

**Keywords:** temporal focusing, multiphoton microscopy, quantum dots, fluorescence microscopy, neurophotonics

## Abstract

The first ever demonstration of temporal focusing with short wave infrared (SWIR) excitation and emission is demonstrated, achieving a penetration depth of 500 *µ*m in brain tissue. This is substantially deeper than the highest previously-reported values for temporal focusing imaging in brain tissue, and demonstrates the value of these optimized wavelengths for neurobiological applications.

## Introduction

Neurophotonic imaging has a challenging set of requirements for new types of instrumentation. The brain exhibits many dynamic phenomena, including electrical activity (which may be probed optically [1]), as well as blood flow [2], and to complicate matters further, many of the most interesting phenomena occur in 3D structures located hundreds of microns below the surface of the *cortex*, or the outermost layer of the brain. It is therefore necessary to image them using a technique that exhibits *axial resolution*, or the ability to unambiguously distinguish light emitted from different *z*-planes.

One example of a technique capable of imaging large areas while maintaining axial resolution is multiphoton microscopy [3]. In multiphoton microscopy, the sample is illuminated by a tightly-focused infrared ultrafast laser pulse composed of photons which, individually, would not have sufficient energy to excite the fluorophore. Nevertheless, when the photon flux is high enough, two (or more) photons can be absorbed simultaneously, their combined energies sufficient to achieve excitation. Because excitation only occurs at the focus of the laser, no out-of-focus emission contributes to the image, thus the system exhibits axial resolution. Nevertheless, raster scanning a spot is slow; to speed the process up, a different form of multiphoton microscopy known as *temporal focusing* can be used to excite the whole field of view in parallel [4, 5].

**Figure 1.**
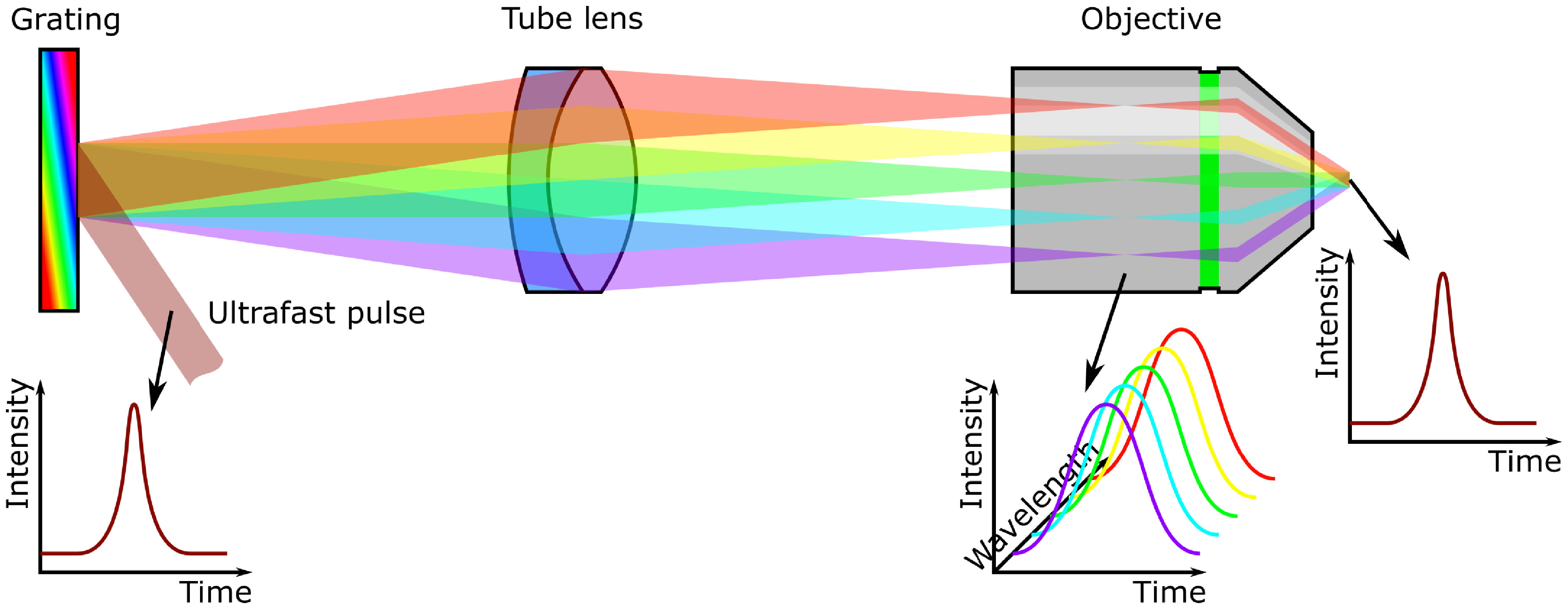
Temporal focusing illustration. An ultrafast pulse strikes a grating and is dispersed; as a consequence, the pulse is spread out in time. The grating surface is imaged onto the sample, thus recombining the pulse components and restoring the optical pulse. The result is that efficient multiphoton excitation can only occur at the focal plane of the objective, and not elsewhere.

Temporal focusing works by using a very high power laser amplifier to illuminate an optical grating (see figure 1). This grating diffracts the beam, and the resulting diffracted light is imaged onto the sample using a microscope tube lens and objective (normally with a dichroic mirror between them). Because an ultrafast pulse is constructed from many different Fourier components (wavelengths) and the grating serves to separate these components from each other, the pulse broadens in time as it propagates from the grating surface. Consequently, multiphoton excitation efficiency drops dramatically, except for where the pulse components are recombined by the objective and tube lens. The result is that fluorophores located over a large area at the focal plane of the objective are efficiently excited, whereas fluorophores above and below this plane are not; or in other words, the microscope exhibits axial resolution.

As mentioned previously, the primary virtue of temporal focusing is that it can excite the whole field of view simultaneously, rather than sequentially as in point-scanning. As a result, it overcomes a difficulty in scaling the field-of-view of a point-scanning system; in point-scanning, there is a tradeoff between per-pixel dwell time, spatial resolution and field of view. In temporal focusing, provided there is sufficient power, the illuminated field of view can be increased arbitrarily, without reducing dwell time or resolution. It should also be noted that the total incident power need not be any higher than for raster scanning; because all points are illuminated simultaneously, the dwell time is equivalent to the exposure time of the frame, rather than the exposure time divided by the number of pixels. As such, even though the instantaneous power density might be substantially lower than for point-scanning, the increase in exposure time makes up for the loss. That said, it is common in temporal focusing systems to increase the field of view at the expense of fluorescence intensity, and as will be described later, this necessitates the use of quantum dots (QDs) to overcome the reduced excitation efficiency.

Like many other optical imaging techniques, temporal focusing has a limited penetration depth in tissue, and while the brain has a comparatively low effective scattering coefficient [6], penetration depths are limited compared to conventional multiphoton excitation. Readers interested in a comparative assessment of temporal focusing and point-scanning multiphoton microscopy are encouraged to read Rowlands *et al* [7]. One approach to increase the tissue penetration depth of temporal focusing is to increase the wavelength of excitation light, since scattering is reduced at longer wavelengths. Even in brain tissue (which is comparatively homogeneous) the scattering mean-free path is approximately 1–2 orders of magnitude lower than the absorption mean-free path [6], hence it is the dominant optical tissue attenuation mechanism. Furthermore, in multiphoton excitation, scattered light contributes to out-of-focus background, whereas absorbed light does not; this limits the contrast of the resulting image, and at a certain point the signal cannot be isolated from the background. This provides an ultimate upper limit on achievable tissue penetration depth, even in the case where infinite laser power is available and sample damage can be ignored.

As a consequence of these phenomena, changing the excitation to longer wavelengths around 1300 nm or 1650 nm (typically referred to as short wave infrared or SWIR wavelengths) is expected to increase the achievable penetration depth of all forms of multiphoton microscopy, temporal focusing included. This is because these wavelengths correspond to regions where absorption due to water is reduced, while still being able to take advantage of reduced tissue scattering [8]. For this study, a 1300 nm excitation wavelength was selected due to the minimal tissue attenuation coefficient described previously.

To perform temporal focusing microscopy with 1300 nm excitation, it is necessary to use a fluorophore with a high multiphoton cross-section at 1300 nm, and an emission wavelength as high as possible, in order to take advantage of reduced scattering while avoiding unwanted one-photon excitation. The need for a high multiphoton cross-section is because temporal focusing typically has reduced irradiance compared to point-by-point scanning, and hence would suffer from poor excitation efficiency if ordinary fluorophores were to be used under conditions of high optical attenuation. Fortunately, these criteria can be adequately addressed by using QDs. These are semiconductor nanoparticles with size-dependent emission spectra, thus allowing almost any arbitrary emission wavelength to be selected just by changing the synthesis conditions. Excitation spectra are broad, extending well into the ultraviolet, and multiphoton cross-sections are very large [9], thus making them ideal probes for multiphoton microscopy. In this study, these dots will be used to label the vasculature in the brain, which is of interest to a number of fields including neurobiology and oncology; brain tumours often induce angiogenesis, and hence can be located by the resulting unusual vascular networks.

To date, there have been no explicit attempts to maximise tissue penetration in brain imaging using temporal focusing microscopy. Nevertheless some example values from the literature include a depth of 100 *µ*m in fixed brain tissue using three-photon structured illumination [10], 50 *µ*m in fixed brain tissue [11], 300 *µ*m in 3D tissue cultured neurons [12], 30 *µ*m for high-speed imaging of *C. elegans* neural activity [13], and a range of values up to 200 *µ*m for different fixed mouse organs [7].

This paper describes the first ever combination of SWIR temporal focusing with custom QDs designed to emit well into the infrared region of the electromagnetic spectrum; a full description is provided of the optical system, fluorophore synthesis and experimental protocols, in order that others may employ these developments in their own work.

## Materials and methods

### Instrument construction

**Figure 2.**
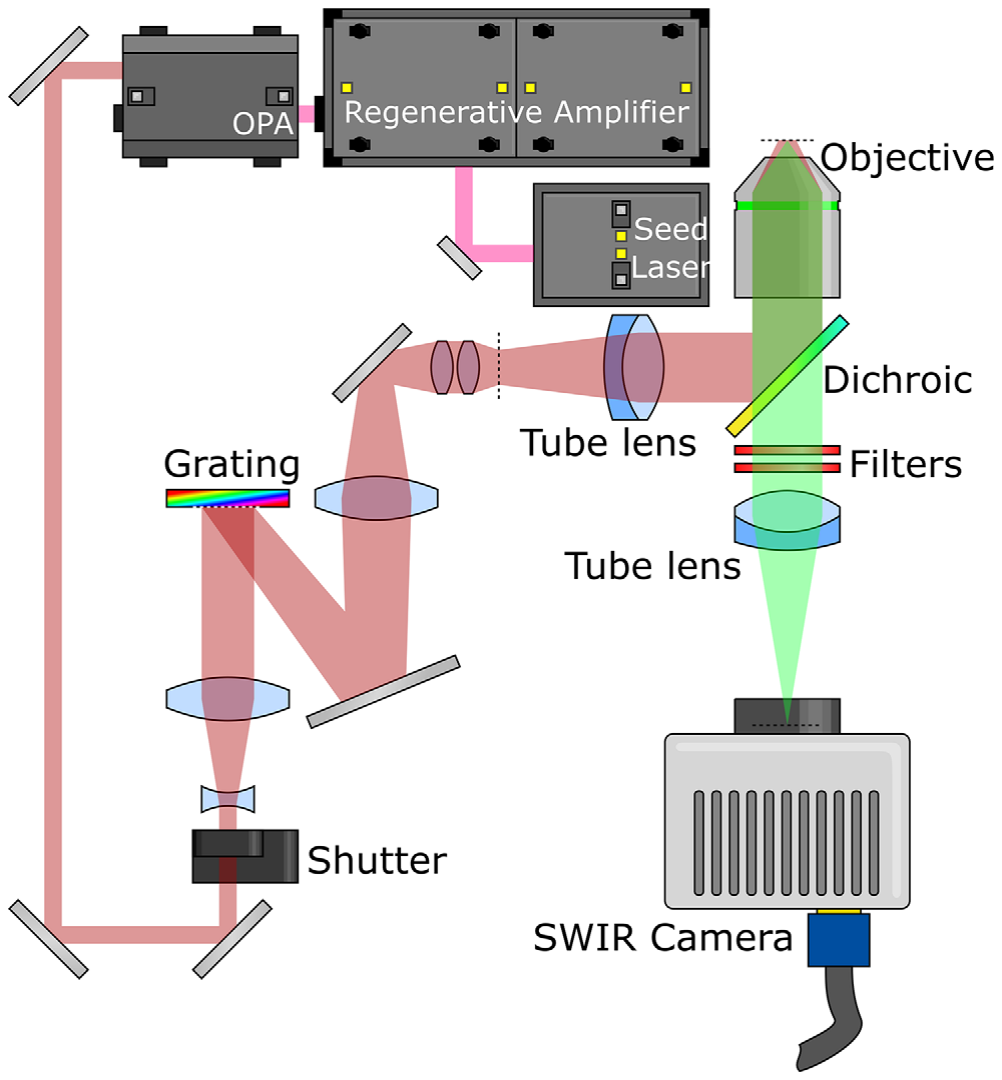
Optical layout. Light from the regenerative amplifier pumps the OPA, which changes the wavelength to 1300 nm. This 1300 nm beam passes through a shutter and beam expander, before striking a grating where it is dispersed. The  −1 order from the grating is demagnified onto an intermediate focal plane (marked with a dotted line) before being imaged onto the sample using a tube lens and microscope objective. Fluorescent emission from the sample then passes through the dichroic mirror and optical filters, forming an axially-resolved image on the camera.

The instrument is based on a temporal focusing design, adapted to operate in the infrared. The design can be seen in figure 2; briefly, 130 fs 800 nm ultrafast optical pulses from a regenerative amplifier (Coherent Legend Elite) are used to pump an optical parametric amplifier (OPA, Coherent Opera Solo) which converts the light to 1300 nm at a repetition rate of 10 kHz. This beam is routed across the optical table, through a periscope and a 3.5  ×  beam expander before striking a custom-made grating (Spectrogon 715.706.410 G 0750 NIR). The  −1 order diffracted from the grating is then demagnified onto the image plane of a microscope (Zeiss Axiovert S100 TV) using a 0.25  ×  telescope. This telescope is made from a 200 mm focal length, 75 mm diameter lens (Edmund Optics 86-923) and a 50 mm focal length compound lens, constructed from two 100 mm focal length 30 mm diameter lenses (Edmund Optics 67-572).

**Figure 3.**
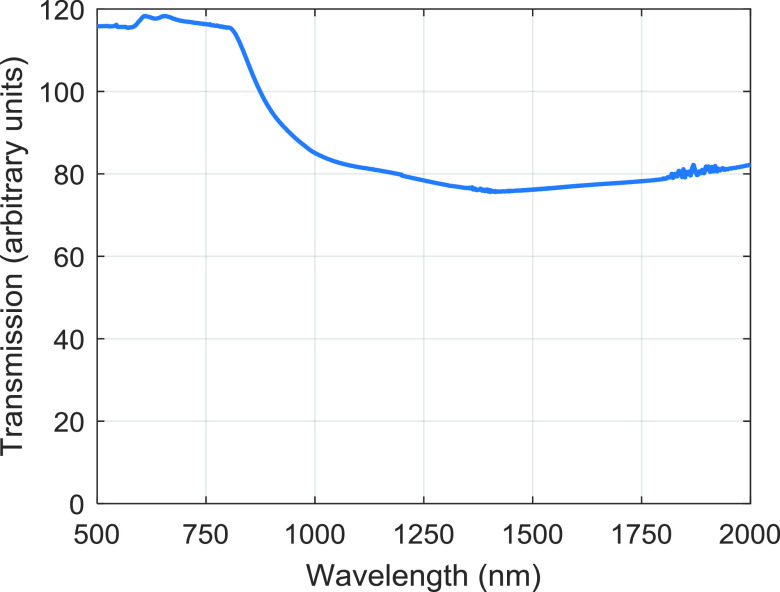
Transmission spectrum for the microscope tube lens. Note that the absolute value for transmission cannot be relied upon; measurements were taken using a commercial spectrometer and the fact that the test object is a lens means the light falling on the detector is a function both of the transmission and the focusing properties of the lens. Relative values between wavelengths are expected to be preserved however.

The image plane is demagnified further by the microscope; the light passes through a 164.5 mm focal length tube lens (Zeiss 425308-0000-000) before being reflected from a 1200 nm short-pass dichroic mirror (Edmund Optics 86-699) and through a microscope objective (Zeiss 421452-9880-000) onto the sample. This objective is well coated for the infrared, with a nominal magnification of 20  ×  and a 1.0 numeric aperture. The objective is mounted on a piezoelectric focusing stage (Piezosystem Jena MIPOS 500 SG), fluorescence light from the sample passes through the dielectric mirror, with residual excitation light rejected by two 1200 nm OD2 short-pass filters (Edmund Optics 86-693). The microscope tube lens then forms the image on the camera (Princeton Instruments NIRvana 640). The transmission spectrum for the microscope tube lens can be seen in figure 3.

### Quantum dot synthesis

InAs-based core–shell–shell quantum dots (QDs) were synthesized according to Franke *et al* [14]. Briefly, indium acetate (4 mmoles) and oleic acid (16 mmoles) were mixed in 1-octadecene (ODE, 20 ml) and degassed at room temperature for 30 min. The mixture was heated to 115 °C for another 60 min, during which a clear solution was formed. The atmosphere was switched to nitrogen and the temperature increased to 295 °C. In a glovebox tris(trimethylgermyl)arsine, (TMGe)_3_As, (0.2 mmoles) was dissolved in trioctylphosphine (TOP, 4 ml) and injected into the solution. After 10 min of reaction time (TMGe)_3_As (0.33 mmoles) in ODE (2 ml) were injected into the solution using a syringe pump and a cannula connecting syringe and flask through a septum. The injection speed was set to 8 ml/h and the reaction was run for 11 min. The heat was removed and the QDs were purified in a glovebox using repeated precipitation-redispersion cycles.

**Figure 4.**
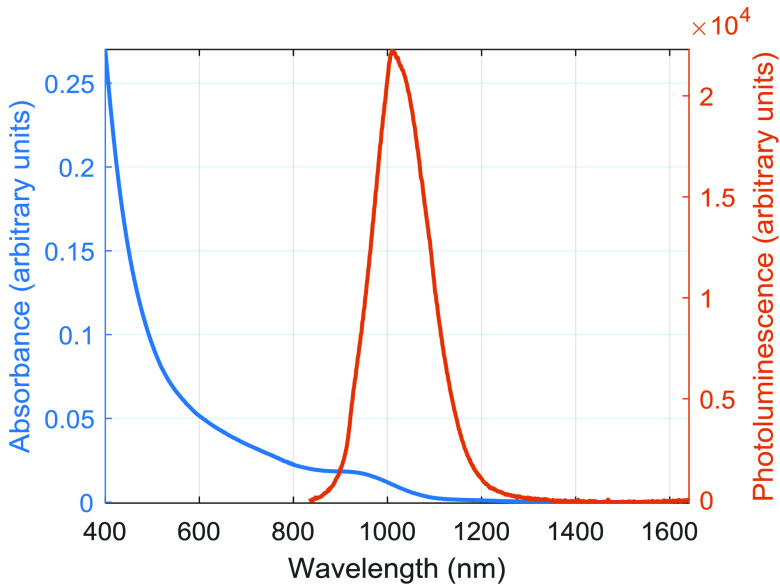
Absorbance and photoluminescence spectra of the QDs.

Roughly 70 nmoles of the purified QDs were redispersed in 2 ml oleylamine and 2 ml ODE. After degassing and heating to 280 °C under nitrogen, the QDs were overcoated with 0.05 M solutions of cadmium oleate and TOP-selenium in ODE, until they exhibited a photoluminescence peak at 1090 nm. After purification through precipitation and redispersion, half the product was dispersed in 1.5 ml ODE and 1.5 ml oleylamine. After degassing and heating to 250 °C under nitrogen, the core–shell QDs were further overcoated using 0.5 M solutions of zinc oleate and ODE-S in ODE. Again, the product was purified through precipitation and redispersion, such that the final core–shell–shell QDs exhibited a photoluminescence peak at 1023 nm (see figure 4) and a quantum yield of 37%.

QDs were transferred into aqueous buffers using a previously reported procedure [2, 15]. Briefly, 1 mg (dry weight) of QDs were mixed with 25 mg of 18:1 PEG2000 PE (1,2-dioleoyl-sn-glycero-3-phosphoethanolamine-N-[methoxy(polyethylene glycol)-2000]) (ammonium salt) (Avanti Polar Lipids, 880130) in chloroform. After brief sonication for 10 s, the solvent was removed under nitrogen flow and 1 ml of isotonic saline or water were added. To completely solubilize the QDs, the aqueous solution was sonicated with a probe sonicator for 5 min and filtered through a 0.2 *µ*m syringe filter.

### Animal procedures

Animal experiments were conducted in accordance with approved institutional protocols of Massachusetts General Hospital and the Massachusetts Institute of Technology. One 8-month-old C57BL/6 mouse was implanted with a cranial window in accordance with a previously-described protocol [16] and allowed several weeks to recover before imaging experiments took place.

### Experimental protocol

The mouse was anesthetized by intraperitoneal injection of ketamine and xylazine. After unconsciousness had been confirmed by a lack of hind leg toe pinch response, a tail vein catheter was placed for injecting the quantum dot solution; the injection was not performed until after the mouse was secured on the microscope.

The mouse was mounted to the microscope with a custom-machined adaptor plate, which was secured to the cranial window supports. The quantum dot solution was then injected, and the vasculature located by imaging with the microscope.

The most superficial fluorescent feature was located, and a focal stack taken by translating the focusing stage by 10 *µ*m per step and taking an image. Exposure duration was 1000 ms for each frame, and a background correction was applied by subtracting the image taken with no incident light. Because the total range of the focussing stage was only 400 *µ*m, it was necessary to re-zero the stage while manually changing the position of the microscope nosepiece in order to continue the focal stack. At certain times the mouse changed position; care was taken to ensure that the focal position did not change, although occasionally some lateral shift was present that was not corrected.

After completion of the experiment and before the mouse recovered from anaesthesia, it was killed with an overdose of ketamine and xylazine.

## Results and discussion

In order to increase the penetration depth of temporal focusing microscopy, a number of technical innovations were necessary. The instrumentation needed to operate at SWIR wavelengths; this included the mirrors, lenses and especially the microscope objective. A Zeiss 421452-9880-000 objective was used, as it is coated for wavelengths up to 1300 nm. In the event that longer wavelength excitation is needed, the Olympus XLPN25XSVMP is available coated up to 1600 nm with only a modest reduction in field of view. In addition, an InGaAs camera was needed to image the QDs, since the quantum efficiency of other silicon-based cameras at the emission wavelength of 1023 nm was poor. Filters were obtained from Edmund Optics as these were the only ones that could be obtained off-the-shelf at an operating wavelength of 1200 nm; because the filters were only specified as OD2, two were used together to achieve the necessary rejection of excitation light.

**Figure 5.**
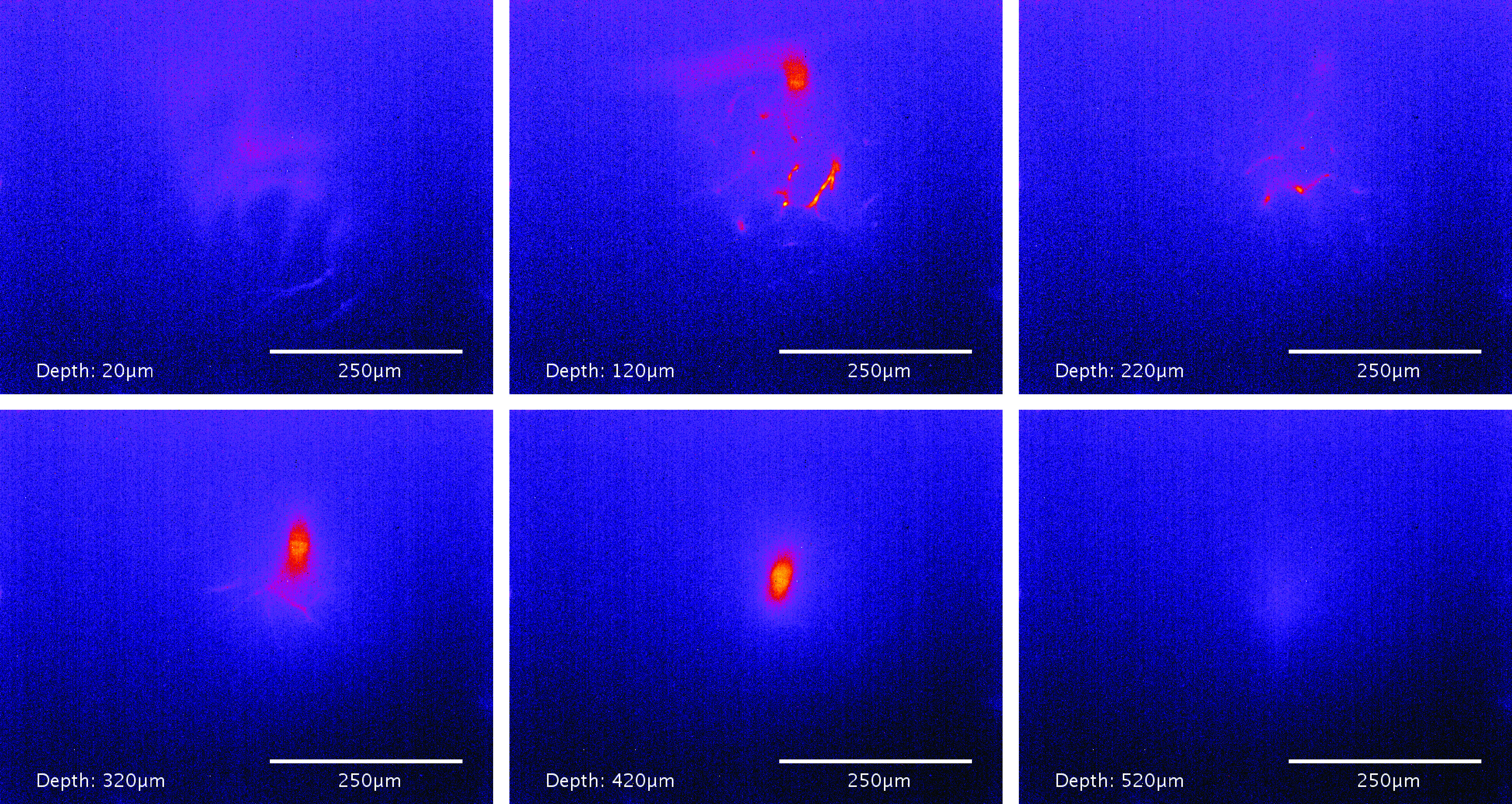
Example frames illustrating the penetration depth of SWIR temporal focusing microscopy. The sample consists of 1023 nm QDs circulating in the brain vasculature of a mouse. The first appearance of a fluorescent feature occurs between 10 *µ*m and 20 *µ*m, and emission is still detectable at 520 *µ*m. The non-uniform background appeared similar in other images, so is not attributed to background fluorescence; it is more likely due to inhomogeneities in the InGaAs sensor noise characteristics (the sensor has a mean read noise of 70*e*^−^), or possibly excitation light passing through the two OD2 filters.

The system achieved a penetration depth of over 500 *µ*m when imaging 1023 nm QDs labelling the largest features in the mouse brain vasculature, which is substantially larger than the 200–300 *µ*m tissue penetration demonstrated in previous studies. This data can be seen in the form of a focal stack as shown in supplementary video 1 (stacks.iop.org/JPhysD/52/264001/mmedia), and example frames from the focal stack can be seen in figure 5. While the finer features in the images are progressively lost as penetration depth increases, larger features can be observed down to a depth of at least 500 *µ*m, and possibly slightly further. Exposure times were all set at 1000 ms, which is limited primarily by noise in the sensor; because InGaAs cameras are regulated within the United States of America under the International traffic in arms regulations (ITAR), high-speed high-resolution and low-noise sensors are unavailable to many researchers (including ourselves). With recent trends towards reduced regulation of these sensors, it is hoped that SWIR temporal focusing can benefit from the improvements in signal-to-noise ratio and readout speed.

## Conclusions

In summary, the first ever temporal focusing system utilizing both SWIR excitation and SWIR imaging was demonstrated, and the achievable penetration depth was substantially improved over previous values from the literature. Achievable frame rates were comparable with fast point-scanning multiphoton microscopes, and given the high read noise of the InGaAs sensor, show potential for substantially faster imaging as and when improved sensors become available to unregulated laboratories. The speed and flexibility of this tool make it very valuable for neurophotonic applications.
